# IdentifICATION OF E3 ubiquitin ligase STUB1 as a negative regulator of FOXP3

**Published:** 2011-01-10

**Authors:** ZJ Chen, ZY Li1, F Lin, ZJ Yao, MI Greene, B Li

**Affiliations:** 1 Key Laboratory of Molecular Virology & Immunology, Institut Pasteur of Shanghai, Chinese Academy of Sciences, PR China; 2 Department of Pathology and Laboratory Medicine, University of Pennsylvania, USA

## 

The level and activity of the forkhead family transcription factor FOXP3 determine the immune function of FOXP3+Tregs. At the beginning of infectious processes, FOXP3+Tregs may regulate effector immune cell responses and lead to failure to control infection. FOXP3+Tregs may also help to limit collateral tissue damage when the antiviral immune responses are too vigorous. Understanding the regulation of FOXP3 and the dynamic ensemble of FOXP3 with enzymatic cofactors in Tregs will provide therapeutic applications for major human viral infectious diseases including HIV, hepatitis B and C viruses.

How FOXP3 protein is negatively regulated in CD4+ regulatory T cells during viral infection and inflammation is currently unknown. Here we report that a stress-signal activated E3 ubiquitin ligase STUB1 appears as a negative regulator of FOXP3. Reciprocal co-immunoprecipitation studies indicate that STUB1 interacts with FOXP3 in vivo. Overexpression of STUB1 specifically promotes the ubiquitination of FOXP3, but not other subfamily transcription factor FOXP1. MG132 treatment increased the ubiquitination level of FOXP3, and overexpression of STUB1 induced ubiquitin-mediated degradation of FOXP3. In contrast to the wild type STUB1, ectopic expression of H260Q mutant STUB1, which disrupts its interaction with E2 conjugation enzymes, didn't lead to FOXP3 degradation. Thus, FOXP3 degradation is mediated by enzymatically active STUB1. Moreover, FOXP3 degradation by STUB1 is also depend on its chaperoned binding, since overexpression of the K30A mutant of STUB1, which is incapable of interacting with chaperone proteins, also fails to promote FOXP3 degradation. Knockdown of endogenous STUB1 by shRNA could increase FOXP3 level in FOXP3 expressing T cells. Functionally, ectopic expression of STUB1 dramatically relieves FOXP3 mediated transcriptional suppression. Our studies identified a novel signal pathway to downregulate FOXP3 activity at posttranslational level by ubiquitin mediated protein degradation.

